# Determinants of effective treatment coverage for posttraumatic stress disorder: findings from the World Mental Health Surveys

**DOI:** 10.1186/s12888-023-04605-2

**Published:** 2023-04-04

**Authors:** Dan J. Stein, Alan E. Kazdin, Richard J. Munthali, Irving Hwang, Meredith G. Harris, Jordi Alonso, Laura Helena Andrade, Ronny Bruffaerts, Graça Cardoso, Stephanie Chardoul, Giovanni de Girolamo, Silvia Florescu, Oye Gureje, Josep Maria Haro, Aimee N. Karam, Elie G. Karam, Viviane Kovess-Masfety, Sing Lee, Maria Elena Medina-Mora, Fernando Navarro-Mateu, José Posada-Villa, Juan Carlos Stagnaro, Margreet ten Have, Nancy A. Sampson, Ronald C. Kessler, Daniel V. Vigo, on behalf of the WHO World Mental Health Survey Collaborators, Sergio Aguilar-Gaxiola, Ali Al-Hamzawi, Yasmin A. Altwaijri, Lukoye Atwoli, Corina Benjet, Guilherme Borges, Evelyn J. Bromet, Brendan Bunting, Jose Miguel Caldas-de-Almeida, Somnath Chatterji, Alfredo H. Cia, Louisa Degenhardt, Koen Demyttenaere, Hristo Hinkov, Chi-yi Hu, Peter de Jonge, Aimee Nasser Karam, Georges Karam, Norito Kawakami, Andrzej Kiejna, Jean-Pierre Lepine, John J. McGrath, Jacek Moskalewicz, Marina Piazza, Kate M. Scott, Tim Slade, Yolanda Torres, Maria Carmen Viana, Harvey Whiteford, David R. Williams, Bogdan Wojtyniak

**Affiliations:** 1 Department of Psychiatry & Mental Health and South African Medical Council Research Unit on Risk and Resilience in Mental Disorders, University of Cape Town and Groote Schuur Hospital, Cape Town, South Africa; 2 Department of Psychology, Yale University, New Haven, CT USA; 3 Department of Psychiatry, University of British Columbia, Vancouver, British Columbia Canada; 4 Department of Health Care Policy, Harvard Medical School, Boston, MA USA; 5 School of Public Health, The University of Queensland, Herston, Queensland Australia; 6 Queensland Centre for Mental Health Research, The Park Centre for Mental Health, Wacol, Queensland Australia; 7 Health Services Research Unit, IMIM-Hospital del Mar Medical Research Institute, CIBER en Epidemiología y Salud Pública (CIBERESP), Pompeu Fabra University (UPF), Barcelona, Spain; 8 Núcleo de Epidemiologia Psiquiátrica - LIM 23, Instituto de Psiquiatria Hospital das Clinicas da Faculdade de Medicina da Universidade de São Paulo, São Paulo, Brazil; 9 Universitair Psychiatrisch Centrum - Katholieke Universiteit Leuven (UPC-KUL), Campus Gasthuisberg, Leuven, Belgium; 10 Lisbon Institute of Global Mental Health, Comprehensive Health Research Center (CHRC)/NOVA Medical School, NOVA University of Lisbon, Lisbon, Portugal; 11 Institute for Social Research, University of Michigan, Ann Arbor, MI USA; 12 IRCCS Istituto Centro San Giovanni di Dio Fatebenefratelli, Brescia, Italy; 13 National School of Public Health, Management and Professional Development, Bucharest, Romania; 14 Department of Psychiatry, University College Hospital, Ibadan, Nigeria; 15 Parc Sanitari Sant Joan de Déu, CIBERSAM, Universitat de Barcelona, Sant Boi de Llobregat, Barcelona, Spain; 16 Institute for Development, Research, Advocacy and Applied Care (IDRAAC), Beirut, Lebanon; 17 Department of Psychiatry and Clinical Psychology, Balamand University, Beirut, Lebanon; 18 Department of Psychiatry and Clinical Psychology, St George Hospital University Medical Center, Beirut, Lebanon; 19 Ecole des Hautes Etudes en Santé Publique (EHESP), EA 4057, Paris Descartes University, Paris, France; 20 Department of Psychiatry, Chinese University of Hong Kong, Tai Po, Hong Kong; 21 National Institute of Psychiatry Ramón de la Fuente Muñiz, Mexico City, Mexico; 22 UDIF-SM, Subdirección General de Planificación, Innovación y Cronicidad, Servicio Murciano de Salud. IMIB-Arrixaca, CIBERESP-Murcia, Murcia, Spain; 23 Colegio Mayor de Cundinamarca University, Faculty of Social Sciences, Bogota, Colombia; 24 Departamento de Psiquiatría y Salud Mental, Facultad de Medicina, Universidad de Buenos Aires, Buenos Aires, Argentina; 25 Trimbos-Instituut, Netherlands Institute of Mental Health and Addiction, Utrecht, Netherlands; 26 Department of Global Health and Social Medicine, Harvard Medical School, Boston, MA USA

**Keywords:** Posttraumatic stress disorder, Contact coverage, Effective treatment coverage, Insurance

## Abstract

**Background:**

Posttraumatic stress disorder (PTSD) is associated with significant morbidity, but efficacious pharmacotherapy and psychotherapy are available. Data from the World Mental Health Surveys were used to investigate extent and predictors of treatment coverage for PTSD in high-income countries (HICs) as well as in low- and middle-income countries (LMICs).

**Methods:**

Seventeen surveys were conducted across 15 countries (9 HICs, 6 LMICs) by the World Health Organization (WHO) World Mental Health Surveys. Of 35,012 respondents, 914 met DSM-IV criteria for 12-month PTSD. Components of treatment coverage analyzed were: (a) any mental health service utilization; (b) adequate pharmacotherapy; (c) adequate psychotherapy; and (d) effective treatment coverage. Regression models investigated predictors of treatment coverage.

**Results:**

12-month PTSD prevalence in trauma exposed individuals was 1.49 (S.E., 0.08). A total of 43.0% (S.E., 2.2) received any mental health services, with fewer receiving adequate pharmacotherapy (13.5%), adequate psychotherapy (17.2%), or effective treatment coverage (14.4%), and with all components of treatment coverage lower in LMICs than HICs. In a multivariable model having insurance (OR = 2.31, 95 CI 1.17, 4.57) and severity of symptoms (OR = .35, 95% CI 0.18, 0.70) were predictive of effective treatment coverage.

**Conclusion:**

There is a clear need to improve pharmacotherapy and psychotherapy coverage for PTSD, particularly in those with mild symptoms, and especially in LMICs. Universal health care insurance can be expected to increase effective treatment coverage and therefore improve outcomes.

**Supplementary Information:**

The online version contains supplementary material available at 10.1186/s12888-023-04605-2.

## Introduction

Posttraumatic stress disorder (PTSD) is a prevalent disorder throughout the world, and is associated with significant morbidity [1, 2]. PTSD leads to individual suffering, reduced quality of life, and considerable societal costs [3, 4]. Fortunately, there is a growing evidence-base of efficacious treatments for this condition, including various forms of psychotherapy and pharmacotherapy [5, 6]. Treatment guidelines for PTSD have been developed by several professional organizations to encourage evidence-based interventions, with most guidelines advocating both pharmacotherapy and psychotherapy as first-line interventions [7, 8]. Data from the WHO World Mental Health Surveys have emphasized that the delay in treatment seeking for mental disorders is a global problem [9], and that there is a treatment gap for a range of these conditions, including anxiety disorders and PTSD [10].

Although contact coverage (the percentage of people in need that get any service) is an important indicator, effective coverage (the percentage that get good care and obtain health benefits) is particularly relevant to health system performance assessment [11, 12]. Determining the extent and predictors of effective coverage for PTSD is an important first step towards developing appropriate strategies to address obstacles to care. While some structural and attitudinal barriers have received attention [13], a number of others, including symptom severity and health insurance have not. The focus on universal health coverage in the Sustainable Developmental Goals further emphasizes the need to investigate effective coverage [14]. A small literature on effective coverage indicators in the area of mental health has emerged, and relies on a number of different methods including need assessment strategies, utilization assessment strategies, and quality assessment strategies [12, 15]. The recent development of an “effective treatment coverage” indicator that quantifies utilization, but also adjusts for quality of care and user adherence, facilitates such work [16].

The WHO World Mental Health Survey Initiative provides a valuable dataset for more detailed investigations of effective treatment coverage across the world, so providing an important foundation for work on addressing key barriers to care and scaling up interventions [16, 17]. We investigated the extent and predictors of treatment coverage for PTSD in individuals who met DSM-IV criteria for 12-month PTSD in a range of high-income countries (HICs) as well as low- and middle-income countries (LMICs). Components of treatment coverage analyzed were: (a) any mental health service utilization; (b) adequate pharmacotherapy; (c) adequate psychotherapy; and (d) effective treatment coverage (adequate severity-specific use of pharmacotherapy and/or psychotherapy).

## Methods

### Sample

The WHO World Mental Health Surveys (WMHS) include 17 community surveys with 35,012 adults across 15 countries, including six classified by the World Bank as low- or middle-income countries (LMICs) and nine classified as high-income countries (HICs) [18]. All samples were based on multi-stage clustered area probability household designs. Samples were nationally representative in 11 surveys, representative of all urbanized areas in two others, and representative of selected regions or Metropolitan areas in the others [18] (Table 1).

**Table 1:** WMH sample characteristics by World Bank income categories^a^Country

	Survey^**b**^	Sample characteristics^**c**^	Field dates	Age range	Sample size		Response rate^**d**^
					Part I	Part II	
**I**. Low and Middle-income countries							
Brazil – São Paulo	São Paulo Megacity	São Paulo metropolitan area	2005–8	18–93	5037	2942	81.3
Colombia	NSMH	All urban areas of the country (approximately 73% of the total national population).	2003	18–65	4426	2381	87.7
Colombia – Medellín	MMHHS	Medellin metropolitan area	2011–12	19–65	3261	1673	97.2
Lebanon	LEBANON	Nationally representative.	2002–3	18–94	2857	1031	70.0
Mexico	M-NCS	All urban areas of the country (approximately 75% of the total national population).	2001–2	18–65	5782	2362	76.6
Nigeria	NSMHW	21 of the 36 states in the country, representing 57% of the national population. The surveys were conducted in Yoruba, Igbo, Hausa and Efik languages.	2002–4	18–100	6752	2143	79.3
Romania	RMHS	Nationally representative.	2005–6	18–96	2357	2357	70.9
Total					(30472)	(14889)	80.1
II. High-income countries							
Argentina	AMHES	Eight largest urban areas of the country (approximately 50% of the total national population)	2015	18–98	3927	2116	77.3
Belgium	ESEMeD	Nationally representative. The sample was selected from a national register of Belgium residents.	2001–2	18–95	2419	1043	50.6
France	ESEMeD	Nationally representative. The sample was selected from a national list of households with listed telephone numbers.	2001–2	18–97	2894	1436	45.9
Germany	ESEMeD	Nationally representative.	2002–3	19–95	3555	1323	57.8
Italy	ESEMeD	Nationally representative. The sample was selected from municipality resident registries.	2001–2	18–100	4712	1779	71.3
Netherlands	ESEMeD	Nationally representative. The sample was selected from municipal postal registries.	2002–3	18–95	2372	1094	56.4
Portugal	NMHS	Nationally representative.	2008–9	18–81	3849	2060	57.3
Spain	ESEMeD	Nationally representative.	2001–2	18–98	5473	2121	78.6
Spain – Murcia	PEGASUS- Murcia	Murcia region. Regionally representative.	2010–12	18–96	2621	1459	67.4
United States	NCS-R	Nationally representative.	2001–3	18–99	9282	5692	70.9
Total					(41104)	(20123)	64.4
III. Total^e^					(71576)	(35012)	70.3

^a^The World Bank (2012) Data. Accessed May 12, 2012 at: http://data.worldbank.org/country. Some of the WMH countries have moved into new income categories since the surveys were conducted. The income groupings above reflect the status of each country at the time of data collection. The current income category of each country is available at the preceding URL

^b^NSMH (The Colombian National Study of Mental Health); MMHHS (Medellín Mental Health Household Study); LEBANON (Lebanese Evaluation of the Burden of Ailments and Needs of the Nation); M-NCS (The Mexico National Comorbidity Survey); NSMHW (The Nigerian Survey of Mental Health and Wellbeing); RMHS (Romania Mental Health Survey); AMHES (Argentina Mental Health Epidemiologic Survey); ESEMeD (The European Study Of The Epidemiology Of Mental Disorders); NMHS (Portugal National Mental Health Survey); PEGASUS-Murcia (Psychiatric Enquiry to General Population in Southeast Spain-Murcia);NCS-R (The US National Comorbidity Survey Replication)

^c^Most WMH surveys are based on stratified multistage clustered area probability household samples in which samples of areas equivalent to counties or municipalities in the US were selected in the first stage followed by one or more subsequent stages of geographic sampling (e.g., towns within counties, blocks within towns, households within blocks) to arrive at a sample of households, in each of which a listing of household members was created and one or two people were selected from this listing to be interviewed. No substitution was allowed when the originally sampled household resident could not be interviewed. These household samples were selected from census area data in all countries other than France (where telephone directories were used to select households) and the Netherlands (where postal registries were used to select households). Several WMH surveys (Belgium, Germany, Italy, Spain-Murcia) used municipal, country resident or universal health-care registries to select respondents without listing households. 10 of the 17 surveys are based on nationally representative household samples

^d^The response rate is calculated as the ratio of the number of households in which an interview was completed to the number of households originally sampled, excluding from the denominator households known not to be eligible either because of being vacant at the time of initial contact or because the residents were unable to speak the designated languages of the survey. The weighted average response rate is 70.3%

^e^The following surveys, included in Thornicroft et al., 2016,^10^ were excluded from this study due to lack of data on the specific drug taken and on adherence to prescribed dosage: Beijing/Shanghai, Bulgaria, Iraq, Israel, Japan, and Peru

Surveys were approved by the review boards of the coordinating organizations, which monitored adherence with procedures for informed consent [19]. Interviews were carried out face-to-face in respondents’ homes by trained lay interviewers. Field training and quality control procedures are described elsewhere [19]. Respondents were aged 18+ in all surveys other than one (19+ in Medellin, Colombia) and had unrestricted upper age limits in most surveys. The average response rate weighted by sample size was 70.3% using the American Association for Public Opinion Research RR1w definition [20].

To reduce respondent burden, interviews were divided into two parts [21]. Part I, administered to all respondents, assessed core mental disorders. Part II assessed additional disorders and correlates and was administered to all respondents with any Part I disorder plus a probability subsample of other Part I respondents. Part II data were weighted to adjust for the under-sampling of Part I non-cases [21]. In total, 71,576 Part I and 35,012 Part II respondents were interviewed. Of these 35,012 respondents, 914 met DSM-IV criteria for 12-month PTSD (Table 2).

**Table 2:** Sociodemographic distribution of the sample by country-income level, among those with 12-month posttraumatic stress disorder

	All countries (***n*** = 914)		High income countries (***n*** = 694)		Low/ middle income countries (***n*** = 220)	
	%/ Mean	(SE)	%/ Mean	(SE)	%/ Mean	(SE)
Gender						
Male	22.7	(1.7)	23.5	(1.8)	20.3	(4.3)
Female	77.3	(1.7)	76.5	(1.8)	79.7	(4.3)
Age Group						
18–29	25.3	(1.9)	22.8	(2.0)	32.7	(4.9)
30–44	31.0	(2.0)	28.9	(2.0)	37.6	(5.0)
45–59	31.8	(2.0)	35.4	(2.2)	21.0	(4.4)
60+	11.9	(1.5)	12.9	(1.9)	8.7	(2.3)
Marital status						
Separated, divorced, or widowed	23.9	(1.7)	26.1	(1.9)	17.0	(3.1)
Never married	22.0	(1.9)	21.8	(2.0)	22.8	(4.5)
Married or cohabitating	54.1	(2.1)	52.1	(2.3)	60.1	(4.5)
Income						
Low	35.0	(2.2)	35.6	(2.5)	32.9	(4.5)
Low-Average	24.1	(1.9)	22.8	(2.0)	28.0	(5.1)
Average-High	23.6	(1.9)	25.0	(2.3)	19.1	(3.7)
High	17.4	(1.8)	16.5	(1.9)	20.0	(4.0)
Education						
Low	20.5	(1.7)	21.2	(2.0)	18.5	(3.4)
Low-Average	35.4	(2.4)	37.4	(2.9)	29.0	(4.6)
Average-High	27.0	(2.0)	24.8	(2.2)	33.6	(4.6)
High	17.2	(1.7)	16.6	(2.0)	19.0	(3.5)
Insurance						
Any Insurance (Yes)	83.9	(1.7)	90.3	(1.3)	64.3	(5.0)
Direct Private/Optional Insurance (Yes)	16.0	(1.7)	20.1	(2.2)	3.4	(1.3)
Employment Status						
Homemaker	13.4	(1.5)	7.6	(1.1)	31.3	(4.4)
Other	20.2	(1.8)	21.5	(2.2)	16.1	(2.9)
Retired	10.5	(1.3)	12.1	(1.6)	5.6	(1.9)
Student	2.4	(0.8)	2.0	(0.8)	3.6	(2.0)
Working	53.5	(2.1)	56.8	(2.3)	43.4	(4.6)
Severity						
Mild	24.0	(2.4)	21.7	(2.6)	31.2	(5.1)
Moderate	35.1	(2.1)	37.8	(2.3)	26.9	(4.2)
Severe	40.9	(2.2)	40.5	(2.6)	41.9	(4.6)
Survey Year^a^						
Continuous	2.9	(0.2)	2.4	(0.2)	4.5	(0.3)

^a^Survey year is continuous, so the mean is shown instead of %

### Measures and data analysis

The interview schedule used in WMH was the WHO Composite International Diagnostic Interview (CIDI) Version 3.0 [22], a fully-structured interview generating lifetime and 12-month prevalence estimates of common DSM-IV disorders that includes stringent protocols of translation, back-translation, expert review, adaptation, and harmonization across sites [23]. Blinded clinical reappraisal interviews with the Structured Clinical Interview for DSM-IV had good concordance with diagnoses based on the CIDI [24]. Respondents with PTSD were considered severe either if their symptoms resulted in severe role impairment (7–10 points) according to the Sheehan Disability Scale [25], moderate if they reported moderate role impairment in the SDS (4–6), and mild if they reported no or moderate role impairment (3 or less).

We classified health treatment providers into two categories: (1) specialist mental health (SMH; psychiatrist, psychologist, other mental health professional in any setting, social worker or counselor in a mental health specialized setting); and (2) general medical (GM; primary care doctor, other medical doctor, any other healthcare professional seen in a GM setting) [18]. Respondents were asked about number of visits with each type of provider in the past 12 months and, for medical providers, about whether they provided psychotherapy, pharmacotherapy, or both. Specific type, dose, and duration were recorded for each psychotropic medication used in the past 12 months. Further details about the treatment variables are presented elsewhere [26].

Consistent with our previous work [18], a series of summary variables was created from these detailed respondent reports. *Contact coverage* involved any 12-month contact with a specialist or general medical provider for a mental health condition. For the pharmacotherapy measures two clinical psychiatrists with expertise in public health (DV, CSW) independently reviewed responses about medications used (which involved selecting from country specific lists including generic and brand names) and classified them. Discrepancies were reconciled by consensus.

As described in our previous work [18], *Adequate medication control* required at least four physician visits [26]. *Medication adherence* required taking the prescribed daily dose at least 90% of the time during the past 12 months of pharmacotherapy (e.g., at least 27 out 30 days in a month) [27–29]. *Adequate pharmacotherapy* required taking an antidepressant with adequate medication control and adherence. While some PTSD guidelines have recommended only specific antidepressants, others have made broader recommendations [7]. A small fraction of people with PTSD may avoid antidepressants due to side effects, failed trials, or other legitimate reasons, so if a non-antidepressant psychotropic was adequately controlled by a psychiatrist with adequate patient adherence, it was also considered adequate.

In congruence with our previous work [30], *Any psychotherapy* required having two or more visits to any specialty mental health provider among help seekers. *Adequate number of sessions* required at least eight sessions. *Adequate psychotherapy* required at least 8 sessions from an adequate provider or still being in treatment after 2 visits. In the case of psychiatrists, for an encounter to be considered as a psychotherapeutic intervention (as opposed to medication adjustment), visits needed to last 30 minutes or more. PTSD guidelines emphasize the efficacy of trauma-focused therapies, but some make more specific recommendations, while others recommend broader classes of psychotherapy [7]. We chose “at least 8 sessions” following the United Kingdom’s National Institute for Health and Care Excellence (NICE) guidelines for the psychotherapy of PTSD [31]; this also has the advantage of mirroring definitions used in previous WMHS research on effective treatment coverage for MDD [18].

We also defined a severity-specific variable for *effective treatment coverage,* which for mild and moderate PTSD required adequate pharmacotherapy and/or adequate psychotherapy, and for severe PTSD both adequate pharmacotherapy and adequate psychotherapy [26, 32]. These criteria are consistent with our previous work on depression. However, the evidence-base on combined treatment for PTSD is thin, and most PTSD guidelines do not recommend initiating treatment with combined pharmacotherapy and psychotherapy [33]. Nevertheless, there is a clinical rationale for considering combined treatment in some patients, and the combination of evidence-based pharmacotherapy and psychotherapy has been recommended when initial treatments fail [34].

The sample for analysis was respondents who met criteria for 12-month PTSD. Differences in within-household probabilities of selection and residual discrepancies between sample and population distributions were adjusted for through weights based on census demographic-geographic variables [21]. The Taylor series linearization method [35] implemented in SUDAAN software [36] was used to estimate standard errors to adjust for weighting and geographic clustering of data. Components of effective treatment coverage were stratified by country-income level.

As described in our previous work [30], bivariate logistic regression analyses were employed to explore significant associations between a broad set of potential predictors (gender, age, marital status, income, education, type of health insurance, private insurance (yes/no), any form of insurance (yes/no), employment status, severity, and survey year) and the outcome of interest, effective treatment coverage for PTSD. A multivariable logistic regression model was employed to predict effective treatment coverage including all the variables that had *p* < .01 in the bivariate analyses. Significance was established at *p* < 0.05, and we report the unadjusted *p* values as well as values adjusted for false discovery rates (FDR) resulting from multiple testing using the Benjamini-Hochberg procedure.

Additionally, as detailed in previous articles in this series [18], for those bivariate models that were significant in predicting effective treatment coverage, we conducted exploratory analyses by decomposing this indicator to identify which components may drive coverage for specific subgroups. Thus, we investigated determinants of contact coverage among those with 12-month PTSD, and of the specific components of treatment (i.e., any pharmacotherapy, adequate pharmacotherapy, any psychotherapy, and adequate psychotherapy) among those with 12-month PTSD and contact coverage. Finally, we stratified the bivariate and multivariable analyses by country-income level.

## Results

### Effective treatment coverage

Twelve-month PTSD prevalence in trauma exposed individuals was 1.49% (S.E., 0.08) across countries. A total of 43.0% (S.E., 2.2) of these cases had contact coverage. Among these individuals with contact coverage (a) 32.7% (S.E., 1.9) received pharmacotherapy, but fewer received antidepressants (22.1% [S.E., 1.6]), and only 13.5% (S.E., 1.4) received adequate pharmacotherapy; (b) 19.9% (S.E., 1.5) received psychotherapy and slightly less (17.2% [S.E., 1.5]) received adequate psychotherapy; (c) 14.4% (S.E., 1.4) received effective treatment coverage (Table 3).

**Table 3:** Coverage for posttraumatic stress disorder by severity

Coverage		Severe ***n*** = 504		Mild/ Moderate ***n*** = 410		Any severity ***n*** = 914		Significance test	
**Numerator**	**Denominator**	**%**	**(SE)**	**%**	**(SE)**	**%**	**(SE)**	**F**	**(*p*-value)**
*Contact coverage*^*a*^	*People with 12-month PTSD* *(n = 914)*	58.1	(2.9)	32.7	(2.6)	43.0	(2.2)	43.31*	(<.001)
*Any psychotropic medication*^*b,c*^		46.5	(2.7)	23.1	(2.3)	32.7	(1.9)	37.64*	(<.001)
*Antidepressants*^*d*^		34.1	(2.5)	13.8	(1.7)	22.1	(1.6)	47.72*	(<.001)
*Adequate medication control*^*e*^		32.1	(2.6)	9.4	(1.5)	18.7	(1.5)	53.48*	(<.001)
*Adequate pharmacotherapy*^*f*^		23.0	(2.4)	7.0	(1.3)	13.5	(1.4)	35.74*	(<.001)
*Any psychotherapy*^*g*^		29.5	(2.5)	13.3	(1.6)	19.9	(1.5)	31.52*	(<.001)
*Adequate psychotherapy*^*h*^		28.0	(2.6)	9.7	(1.5)	17.2	(1.5)	47.56*	(<.001)
*Effective coverage*^*i*^		18.5	(2.2)	11.7	(1.7)	14.4	(1.4)	6.03*	(0.01)

*Abbreviations*: *PTSD* Posttraumatic stress disorder, *SE* Standard error

*Significant at the .05 level, two-sided test

^a^Contact coverage required any 12-month contact with a specialist or general medical provider for a mental health condition

^b^Requires any 12-month healthcare/contact coverage too

^c^Any psychotropic required receiving any psychotropic and any 12-month healthcare

^d^Antidepressants required appropriate medication (antidepressant) and any 12-month healthcare

^e^Adequate medication control required at least four physician visits

^f^Adequate pharmacotherapy required taking an antidepressant with adequate medication control and adherence

^g^Any psychotherapy required having two or more visits to any specialty mental health provider among help seekers

^h^Adequate psychotherapy required at least 8 sessions from an adequate provider or still being in treatment after 2 visits

^i^Effective treatment coverage, for mild and moderate PTSD required adequate pharmacotherapy and/or adequate psychotherapy, and for severe PSTD both adequate pharmacotherapy and adequate psychotherapy

**Fig. 1:**
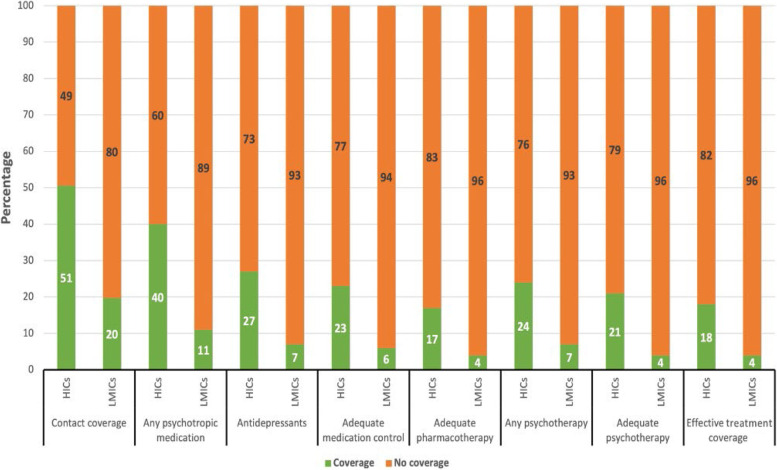
Treatment coverage for posttraumatic stress disorder (12-month PTSD). HICs (*n* = 694): High income countries; LMICs (*n* = 220): Low/ middle income countries. *Contact coverage* required any 12-month contact with a specialist or general medical provider for a mental health condition. *Any psychotropic* required receiving any psychotropic and any 12-month healthcare. A*ntidepressants* required appropriate medication (antidepressant) and any 12-month healthcare. *Adequate medication control required at least four physician visits*. *Adequate pharmacotherapy* required taking an antidepressant with adequate medication control and adherence. *Any psychotherapy* required having two or more visits to any specialty mental health provider among help seekers. *Adequate psychotherapy* required at least 8 sessions from an adequate provider or still being in treatment after 2 visits. *Effective treatment coverage,* for mild and moderate PTSD required adequate pharmacotherapy and/or adequate psychotherapy, and for severe PSTD both adequate pharmacotherapy and adequate psychotherapy

Stratification by country income-level (HIC vs LMIC) demonstrated that (a) contact coverage (50.6% vs 19.8%; (b) adequate pharmacotherapy (16.6% vs 4.1%); (c) adequate psychotherapy (21.3% vs 4.5%; and (d) effective treatment coverage (17.8% vs 4.1%) were all higher in HICs than in LMICs (Fig. 1).

### Predictors of effective treatment coverage

In initial bivariate models, level of education, type of insurance, and severity of symptoms were associated with effective treatment coverage (Table 4). Those with low-average and average-high levels of education were less likely to receive effective treatment than those with high level of education. In general, those with any form of insurance are more likely to receive effective treatment coverage than those with no insurance. Having state funded coverage or subsidized insurance made it more likely to receive any modality of therapy and effective treatment, while those with insurance through employment or national social security were more likely to receive any pharmacotherapy, adequate pharmacotherapy, or effective treatment. Those with mild or moderate symptoms were less likely to receive any or adequate pharmacotherapy, or any or adequate psychotherapy, and those with mild symptoms were less likely to receive effective treatment. Stratification by country-income level showed similar findings in HICs (Supplemental Tables S1 and S2), while in LMICs the sample size did not allow for analyses by effective treatment and its components, analyses of contact coverage found that any form of insurance was particularly important in predicting contact coverage (Supplement Table S3).

**Table 4:** Bivariate predictors of effective coverage and its components among those with 12-Month posttraumatic stress disorder, in all countries (*n* = 914)^a^

	Among those with 12-month PTSD (***n*** = 914), received contact coverage^**b**^															Among those with 12-month PTSD (***n*** = 914), received effective coverage^**g**^			
				Received any pharmacotherapy			Received adequate pharmacotherapy^**d**^			Received any psychotherapy^**e**^			Received adequate psychotherapy^**f**^						
	OR	(95% CI)	F test	OR	(95% CI)	F test	OR	(95% CI)	F test	OR	(95% CI)	F test	OR	(95% CI)	F test	OR	(95% CI)	F test	FDR^h^
**Level of education**																			
Low	0.9	(0.6–1.5)	2.3^@^	1.1	(0.7–1.9)	5.3^*^	0.9	(0.5–1.7)	3.0*	0.6	(0.4–1.1)	1.7	0.6	(0.3–1.1)	1.5	0.7	(0.4–1.3)	2.3^@^	0.07
Low-Average	0.6	(0.4–1.1)		0.7	(0.4–1.1)		0.6	(0.3–1.1)		0.6*	(0.4–0.99)		0.6	(0.4–1.1)		0.5*	(0.3–0.9)		
Average-High	0.5*	(0.3–0.9)		0.5*	(0.3–0.7)		0.5*	(0.3–0.8)		0.6	(0.4–1.04)		0.6	(0.4–1.3)		0.5*	(0.3–0.9)		
High	REF			REF			REF			REF			REF			REF			
**Type of insurance**																			
*No insurance coverage*	REF			REF			REF			REF			REF			REF			
State funded coverage or subsidized insurance	3.9*	(2.2–6.9)		3.5*	(1.6–7.9)		3.5*	(1.5–8.3)		2.7*	(1.3–5.6)		3.6*	(1.5–8.6)		3.1*	(1.4–7.1)		
Other	1.8*	(1.01–3.2)		1.8	(0.9–3.5)		1.4	(0.4–3.3)		1.5	(0.8–2.8)		1.8	(0.8–4.1)		1.6	(0.8–3.3)		
Direct Private/Optional Insurance	1.9	(0.8–4.2)	6.7*	1.6	(0.6–4.3)	4.4*	1.7	(0.6–5.2)	3.8*	1.7	(0.7–4.1)	3.9*	1.8	(0.6–5.0)	3.1*	2.2	(0.8–6.1)	2.7*	0.06
Insurance through employment or national social security	5.9*	(1.7–20.2)		5.4*	(1.5–19.0)		8.3*	(1.7–41.5)		3.4	(0.95–11.9)		2.9	(0.7–11.6)		5.4*	(1.4–21.4)		
**Insurance**																			
Any Insurance (Yes)	2.3*	(1.3–4.1)	8.5*	2.2*	(1.1–4.4)	4.8*	2.1	(0.9–4.7)	3.1^@^	1.8	(0.97–3.4)	3.5	2.2*	(1.01–4.9)	4.0*	2.1*	(1.02–4.5)	4.1*	0.06
**Severity**																			
Mild	0.2*	(0.1–0.3)		0.1*	(0.08–0.2)		0.1*	(0.04–0.3)		0.3*	(0.2–0.6)		0.2*	(0.08–0.3)		0.4*	(0.2–0.8)		
Moderate	0.4*	(0.3–0.6)	29.7*	0.5*	(0.3–0.7)	24.9*	0.3*	(0.2–0.5)	18.0*	0.4*	(0.3–0.6)	15.9^*^	0.3*	(0.2–0.5)	25.0*	0.8	(0.5–1.2)	3.7*	0.06
Severe	REF			REF			REF			REF			REF			REF			

*Abbreviations*: *PTSD* Posttraumatic stress disorder, *OR* Odds ratio, *CI* Confidence interval

*Significant at the .05 level, two-sided test ^@^ *P* < 0.1

^a^Models are bivariate with each demographic predictor in separate models, controlling for country dummies. The following variables were non-significant or *P* > 0.1: age, sex, marital status, income, employment status and survey year

^b^Contact coverage required any 12-month contact with a specialist or general medical provider for a mental health condition

^c^Any psychotropic required receiving any psychotropic and any 12-month healthcare

Antidepressants required appropriate medication (antidepressant) and any 12-month healthcare

Adequate medication control required at least four physician visits

^d^Adequate pharmacotherapy required taking an antidepressant with adequate medication control and adherence

^e^Any psychotherapy required having two or more visits to any specialty mental health provider among help seekers

^f^Adequate psychotherapy required at least 8 sessions from an adequate provider or still being in treatment after 2 visits

^g^Effective treatment coverage, for mild and moderate PTSD required adequate pharmacotherapy and/or adequate psychotherapy, and for severe PSTD both adequate pharmacotherapy and adequate psychotherapy

^h^FDR: False discovery rate adjustment for multiple testing implementing the Benjamini-Hochberg method

In the final multivariable model, after adjusting for the FDR, any form of insurance (OR = 2.31, 95% CI 1.17, 4.57) and mild symptom severity (OR = .35, 95% CI 53,1.08) remained significant predictors (Table 5). Stratification by country-income level showed similar findings in HICs (Supplement Table S2), while in LMICs although sample size again did not allow analyses by effective treatment and its components any form of insurance was again particularly important in predicting contact coverage (Supplement Table S3).

**Table 5:** Multivariable model of effective coverage among those with 12-Month posttraumatic stress disorder, in all countries (*n* = 914) ^a^

	Among those with 12-month PTSD (***n*** = 914), received effective coverage			
	OR	(95% CI)	F test	FDR^b^
**Level of education**				
Low-Average Education Y/N	0.76	(0.52–1.11)	2.02	0.157
**Type of insurance**				
Any Insurance Y/N	2.31*	(1.17–4.57)	5.88*	0.025
**Severity**				
Mild	0.35*	(0.18–0.70)		
Moderate	0.76	(0.53–1.08)	5.10*	0.021
Severe	REF			
**Global F test for multivariate model**			7.08*	

*Abbreviations*: *PTSD* Posttraumatic stress disorder, *O*, Odds ratio, *CI* Confidence interval

*Significant at the .05 level, two-sided test

^a^Model is a multivariate model with all rows in the same model, controlling for country dummies

^b^FDR: False discovery rate adjustment for multiple testing implementing the Benhamini-Hochberg method

## Discussion

Key findings from this analysis of WHO World Mental Health Surveys (WMHS) data were 1) that only 43.0.% of those with 12-month PTSD had contact coverage, with fewer receiving adequate pharmacotherapy (13.5%), adequate psychotherapy (17.2%), or effective treatment coverage (adequate severity specific use of pharmacotherapy and/or psychotherapy) (14.4%), and with all components of treatment coverage lower in LMICs than HICs, and 2) that lack of insurance and mild clinical symptoms were predictive of lower effective treatment coverage for PTSD.

The literature on treatment coverage of PTSD is relatively sparse. In veterans in the United States, studies have found that 23–40% of those who screened positive for a mental health issue received professional assistance [37], that 53% of those recently diagnosed with PTSD in primary care started treatment at that level [38], and that only 33% of veterans have received minimally adequate PTSD care [39]. In earlier work from the WMHS, of those with a 12-month anxiety disorder or PTSD, only 41.3% perceived a need for care, and only 27.6% received any treatment [10].

Several barriers to treatment of PTSD have previously been reported in the literature. These include both structural barriers such as lack of those providing evidence-based psychotherapy for PTSD [40], and attitudinal barriers such as ambivalence about treatment seeking [41]. In veterans in the US, those recently diagnosed with and treated at primary care level are more likely to receive pharmacotherapy [42]. In earlier work from the WMHS on barriers to care, low perceived need was the most common reason for not initiating treatment and was more common among moderate and mild than severe cases. Notably, attitudinal barriers dominated for mild-moderate cases, while structural barriers were more important for severe cases [13].

The finding that patients with more severe symptoms are more likely to receive effective treatment coverage suggests that a more comprehensive treatment package is available for people who suffer severe PTSD, compared to those that suffer severe MDD [18]. While more severe PTSD symptoms may be associated with more disability, previous findings from WMHS have emphasized the graded relationship between PTSD severity and clinical outcomes [43]. Thus decisions about treating cases should be based on cost-effectiveness rather than severity [44]. There is growing evidence of the cost-effectiveness of interventions for individuals meeting diagnostic criteria for PTSD, although further such work is needed [4].

The most important social determinant of treatment coverage was the presence of insurance. Private insurance was also found to be a significant predictor in our previous work on effective treatment coverage for major depressive disorder, but in this case the difference is more salient: every form of insurance warrants increased coverage for PTSD when compared to no insurance [18]. A focus on the relevance of insurance for treatment coverage is timely given the current emphasis on universal health care coverage [14, 45].

Some limitations deserve emphasis. First, the data regarding service utilization and adherence are dependent on respondent recall. However, the focus here on 12-month treatment rather than lifetime prevalence minimizes recall bias. To compensate for potential bias we used a particularly stringent compliance threshold (taking the indicated dose at least 90% of the time) [27–29]. With respect to the time-span covered by surveys, our models included dummy control variables for each survey, an approach that controls for survey year, so that findings are based on pooled within-survey results. Second, several aspects of the treatment provided, such as adherence to treatment manuals, may influence judgments of whether or not treatment coverage was effective. While a clinical trial allows assessment of such issues, it does not have the statistical power of an epidemiological approach. Third, our definitions of adequate treatment mirror our prior work on depression, but the evidence-base of randomized controlled trials of interventions for PTSD is smaller, with fewer approved pharmacotherapies, fewer evidence-based psychotherapies, and less evidence for the value of combined pharmacotherapy and psychotherapy [33]. Although our definitions of adequate treatment overlap in part with evidence-based guidelines for PTSD such as the NICE guideline their limitations deserve emphasis; for example, although such treatment guidelines for PTSD note the value of both pharmacotherapy and psychotherapy, they emphasize initiating treatment with either specific antidepressants or psychotherapies, rather than their combination.

In summary, these data emphasize that there is a clear need to improve pharmacotherapy and psychotherapy coverage for PTSD, particularly in those with mild symptoms, and especially in LMIC contexts. Previous work has emphasized the potential value of increasing human resources for mental health care and of increasing population mental health literacy in order to address structural and attitudinal barriers to accessing mental health services [14]. A key component of addressing such barriers is the provision of universal health care insurance for both physical and mental disorders.

## Supplementary Information

**Additional file 1: Supplemental Table 1.** Bivariate predictors of effective coverage and its components among those with 12-Month posttraumatic stress disorder, in HICs countries (*n*=694)^a^. **Supplemental Table 2.** Multivariable model of effective coverage among those with 12-Month posttraumatic stress disorder, in high-income countries (*n*=694) ^a^. **Supplemental Table 3.** Predictors of contact coverage among those with 12-Month posttraumatic stress disorder, in LMICs countries (*n*=220) ^a^.

## Data Availability

Access to the cross-national World Mental Health (WMH) data is governed by the organizations funding and responsible for survey data collection in each country. These organizations made data available to the WMH consortium through restricted data sharing agreements that do not allow us to release the data to third parties. The exception is that the U.S. data are available for secondary analysis via the Inter-University Consortium for Political and Social Research (ICPSR), http://www.icpsr.umich.edu/icpsrweb/ICPSR/series/00527.
