# Supplementary material for: Determinants of effective treatment coverage for posttraumatic stress disorder: findings from the World Mental Health Surveys
Source: BMC Psychiatry. 2023 Apr 4;23:226. doi: 10.1186/s12888-023-04605-2 (PMC10074702; doi:10.1186/s12888-023-04605-2)
Supplement: Supplementary file 1 — Additional file 1: Supplemental Table 1. Bivariate predictors of effective coverage and its components among those with 12-Month posttraumatic stress disorder, in HICs countries (n=694)a. Supplemental Table 2. Multivariable model of effective coverage among those with 12-Month posttraumatic stress disorder, in high-income countries (n=694) a. Supplemental Table 3. Predictors of contact coverage among those with 12-Month posttraumatic stress disorder, in LMICs countries (n=220) a. [file 12888_2023_4605_MOESM1_ESM.docx]

| **Supplementary Tables** | | | | | | | | | | | | | | | | | | | | | | | | | | | | | | | | |  |
| --- | --- | --- | --- | --- | --- | --- | --- | --- | --- | --- | --- | --- | --- | --- | --- | --- | --- | --- | --- | --- | --- | --- | --- | --- | --- | --- | --- | --- | --- | --- | --- | --- | --- |
|  | | | | | | | | | | | | | | | | | | | | | | | | | | | | | | | | |  |
|  | | | | | | | | | | | | | | | | | | | | | | | | | | | | | | | | |  |
| **Supplemental Table 1. Bivariate predictors of effective coverage and its components among those with 12-Month posttraumatic stress disorder, in HICs countries (n=694)**^a^ | | | | | | | | | | | | | | | | | | | | | | | | | | | | | | | | |  |
|  | | | | | | | | | | | | | | | | | | | | | | | | | | | | | | | | |  |
|  | **Among those with 12-month PTSD (n=694), received contact coverage** | | | |  | | | | | | | | | | | | | | | | | | | | **Among those with 12-month PTSD (n=914), received effective coverage** | | | | | | |  |  |
|  |  |  |  |  | **Received any pharmacotherapy** | | | | | **Received adequate pharmacotherapy** | | | | | | **Received any psychotherapy** | | | | | **Received adequate psychotherapy** | | | |  |  |  |  |  |  |  |  |  |
|  | **OR** | **(95% CI)** | **F test** | | **OR** | | **(95% CI)** | **F test** | | **OR** | | **(95% CI)** | | **F test** | | **OR** | | **(95% CI)** | **F test** | | **OR** | | **(95% CI)** | **F test** | **OR** | | **(95% CI)** | **F test** | | **FDR**^b^ | |  |  |
| **Level of education** |  |  |  | |  | |  |  | |  | |  | |  | |  | |  |  | |  | |  |  |  | |  |  | |  | |  |  |
| Low | 0.8 | (0.5-1.3) | 2.3^@^ | | 0.96 | | (0.6-1.7) | 4.1^*^ | | 1.0 | | (0.5-1.9) | | 3.4* | | 0.7 | | (0.4-1.3) | 1.2 | | 0.6 | | (0.3-1.1) | 1.3 | 0.7 | | (0.3-1.3) | 2.6^@^ | | 0.07 | |  |  |
| Low-Average | 0.5* | (0.3-0.9) |  | | 0.6* | | (0.3-0.98) |  | | 0.5* | | (0.3-0.99) | |  | | 0.6 | | (0.4-1.02) |  | | 0.6 | | (0.4-1.03) |  | 0.5* | | (0.3-0.9) |  | |  | |  |  |
| Average-High | 0.5* | (0.3-0.9) |  | | 0.5* | | (0.3-0.8) |  | | 0.5* | | (0.3-0.8) | |  | | 0.7 | | (0.4-1.2) |  | | 0.7 | | (0.4-1.1) |  | 0.5* | | (0.3-0.9) |  | |  | |  |  |
| High | REF |  |  | | REF | |  |  | | REF | |  | |  | | REF | |  |  | | REF | |  |  | REF | |  |  | |  | |  |  |
| **Type of insurance** |  |  |  | |  | |  |  | |  | |  | |  | |  | |  |  | |  | |  |  |  | |  |  | |  | |  |  |
| *No insurance coverage* | REF |  |  | | REF | |  |  | | REF | |  | |  | | REF | |  |  | | REF | |  |  | REF | |  |  | |  | |  |  |
| State funded coverage or subsidized insurance | 3.4* | (1.7-6.9) |  | | 3.6* | | (1.5-8.3) |  | | 3.2* | | (1.4-7.4) | |  | | 2.8* | | (1.3-5.9) |  | | 3.6* | | (1.5-8.7) |  | 2.9* | | (1.3-6.7) |  | |  | |  |  |
| Other | 1.5 | (0.8-2.7) |  | | 1.6 | | (0.8-3.2) |  | | 1.2 | | (0.6-2.6) | |  | | 1.3 | | (0.7-2.5) |  | | 1.6 | | (0.7-3.7) |  | 1.3 | | (0.7-2.7) |  | |  | |  |  |
| Direct Private/Optional Insurance | 1.6 | (0.6-3.9) | 5.9* | | 1.5 | | (0.5-4.2) | 4.1* | | 1.6 | | (0.5-4.8) | | 4.2* | | 1.6 | | (0.6-4.1) | 4.5* | | 1.6 | | (0.6-4.8) | 3.7* | 2.0 | | (0.7-5.7) | 2.9* | | 0.07 | |  |  |
| Insurance through employment or national social security | 4.8* | (1.01-22.9) |  | | 5.5* | | (1.2-24.6) |  | | 9.8* | | (1.8-53.4) | |  | | 2.4 | | (0.6-8.9) |  | | 2.3 | | (0.5-10.2) |  | 5.3* | | (1.2-22.4) |  | |  | |  |  |
| **Insurance** |  |  |  |  | |  | |  |  | |  | |  | |  | |  | |  |  | |  | |  |  |  | | |  | |  | | |
| Any Insurance (Yes) | 1.9* | (1.1-3.4) | 4.8* | 2.0 | | (0.96-4.2) | | 3.5^@^ | 1.8 | | (0.8-4.0) | | 2.2 | | 1.7 | | (0.9-3.2) | | 2.4 | 2.0 | | (0.9-4.4) | | 2.9^@^ | 1.9 | (0.9-3.8) | | | 2.8^@^ | | 0.10 | | |
| **Severity** |  |  |  |  | |  | |  |  | |  | |  | |  | |  | |  |  | |  | |  |  |  | | |  | |  | | |
| Mild | 0.2* | (0.1-0.3) | 26.5* | 0.1* | | (0.07-0.2) | |  | 0.1* | | (0.04-0.4) | |  | | 0.3* | | (0.1-0.5) | |  | 0.2* | | (0.07-0.3) | |  | 0.4* | (0.2-0.8) | | |  | |  | | |
| Moderate | 0.5* | (0.3-0.7) |  | 0.5* | | (0.3-0.7) | | 22.8* | 0.3* | | (0.2-0.6) | | 15.1* | | 0.4* | | (0.3-0.6) | | 16.1^*^ | 0.4* | | (0.2-0.6) | | 23.7* | 0.8 | (0.5-1.3) | | | 3.4* | | 0.07 | | |
| Severe | REF |  |  | REF | |  | |  | REF | |  | |  | | REF | |  | |  | REF | |  | |  | REF |  | | |  | |  | | |
|  |  |  |  |  | |  | |  |  | |  | |  | |  | |  | |  |  | |  | |  |  |  | | |  | |  | | |

Abbreviations. PTSD, posttraumatic stress disorder; OR, odds ratio; CI; confidence interval

*Significant at the .05 level, two-sided test ^@^ P<0.1.

^a^Models are bivariate with each demographic predictor in separate models, controlling for country dummies. The following variables were non-significant or P>0.1: age, sex, marital status, income, employment status and survey year

^b^FDR: False discovery rate adjustment for multiple testing implementing the Benjamini-Hockberg method

*Contact coverage* required any 12-month contact with a specialist or general medical provider for a mental health condition

*Any psychotropic* required receiving any psychotropic and any 12onth healthcare

A*ntidepressant* required appropriate medication (antidepressant) AND any 12-month healthcare

*Adequate medication control required at least four physician visits*

*Adequate pharmacotherapy* required taking an antidepressant with adequate medication control and adherence

*Any psychotherapy* required having two or more visits to any specialty mental health provider among help seekers.

*Adequate psychotherapy* required at least 8 sessions from an adequate provider or still being in treatment after 2 visits

*Effective treatment coverage,* for mild and moderate PTSD required adequate pharmacotherapy and/or adequate psychotherapy, and for severe PSTD both adequate pharmacotherapy and adequate psychotherapy

| **Supplemental Table 2. Multivariable model of effective coverage among those with 12-Month posttraumatic stress disorder, in high-income countries (n=694)** ^a^ | | | | | | |
| --- | --- | --- | --- | --- | --- | --- |
|  | | | | | | |
|  | ***Among those with 12-month PTSD (n=694), received effective coverage*** | | | | | |
|  | ***OR*** | ***(95% CI)*** |  | ***F test*** | | ***FDR****^b^* |
| **Level of education** |  |  |  |  | |  |
| Low-Average Education Y/N | 0.73 | (0.50-1.06) |  | 2.89 | | 0.092 |
| **Type of insurance** |  |  |  |  | |  |
| Any Insurance Y/N | 2.11* | (1.07-4.16) |  | 4.7* | | 0.048 |
| **Severity** |  |  |  |  | |  |
| Mild | 0.33* | (0.16-0.68) |  |  | |  |
| Moderate | 0.81 | (0.56-1.17) |  | 4.64* | | 0.035 |
| Severe | REF |  |  |  |  |  |
| **Global F test for multivariate model** |  |  |  | 6.33* | |  |
|  |  |  |  |  |  | |

Abbreviations. PTSD, posttraumatic stress disorder; OR, odds ratio; CI; confidence interval

*Significant at the .05 level, two-sided test.

^a^Model is a multivariate model with all rows in the same model, controlling for country dummies.

^b^FDR: False discovery rate adjustment for multiple testing implementing the Benhamini-Hockberg method

| **Supplemental Table 3. Predictors of contact coverage among those with 12-Month posttraumatic stress disorder, in LMICs countries (n=220)** ^a^ | | | | | | | | | | | | |  |
| --- | --- | --- | --- | --- | --- | --- | --- | --- | --- | --- | --- | --- | --- |
|  | **Bivariate: among those with 12-month PTSD (n=220),**  **received contact coverage** | | | | | | **Multivariate: among those with 12-month PTSD (n=220),**  **received contact coverage** | | | | | |  |
|  | **OR** | **(95% CI)** | **F test** | ***FDR****^b^* | | | **OR** | **(95% CI)** | | | **F test** | ***FDR****^b^* | |
| **Level of education** |  |  |  |  | | |  |  | | |  |  | |
| Low | 1.8 | (0.4-7.7) |  |  | | | - | - | | |  |  | |
| Low-Average | 1.9 | (0.4-7.8) | 1.47 | 0.22 | | | 1.59 | (0.6-3.9) | | | 1 1.06 | 0.31 | |
| Average-High | 0.7 | (0.1-3.4) |  |  | | | - | - | | |  |  | |
| High | REF |  |  |  | | | - | - | | |  |  | |
| **Type of insurance** |  |  |  |  | | |  |  | | |  |  | |
| *No insurance coverage* | REF |  |  |  | | | REF |  | | |  |  | |
| State funded coverage or subsidized insurance | 13.6* | (2.3-81.1) |  |  | | | - | - | | | - |  | |
| Other | 11.3* | (1.8-72.8) |  |  | | | - | - | | | - |  | |
| Direct Private/Optional Insurance | 5.5 | (0.8-37.1) | 2.69* | 0.04 | | | - | - | | | - |  | |
| Insurance through employment or national social security | 35.4* | (3.4-363.3) |  |  | | | - | - | | | - |  | |
| **Insurance** |  |  |  | |  |  | | |  |  | |  | |
| Any Insurance (Yes) | 12.3* | (2.3-64.9) | 8.83* | | 0.008 | 11.2* | | | (8.0-15.7) | 205.34* | | <0.0001 | |
| **Severity** |  |  |  | |  |  | | |  |  | |  | |
| Mild | 0.3* | (0.1-0.9) | 5.57* | | 0.008 | 0.4* | | | (0.2-0.6) |  | |  | |
| Moderate | 0.3* | (0.1-0.7) |  | |  | 0.3* | | | (0.1-0.6) | 11.37* | | <0.0001 | |
| Severe | REF |  |  | |  | REF | | |  |  | |  | |
|  |  |  |  | |  |  | | |  |  | |  | |

Abbreviations. PTSD, posttraumatic stress disorder; OR, odds ratio; CI; confidence interval

*Significant at the .05 level, two-sided test ^@^ P<0.1.

^a^Models are bivariate with each demographic predictor in separate models, controlling for country dummies. The following variables were non-significant or P>0.1: age, sex, marital status, income, employment status and survey year

*^b^Contact coverage* required any 12-month contact with a specialist or general medical provider for a mental health condition

^c^FDR: False discovery rate adjustment for multiple testing implementing the Benjamini-Hockberg method
